# Consulting “Dr. Google”: how the digital search for internet health information influences doctor-patient relationship

**DOI:** 10.1590/0102-311XEN153623

**Published:** 2025-08-08

**Authors:** Ricardo Moyses de Arruda, Ibrahim Ali Ayoub, Rui Nunes, Raymundo Soares de Azevedo, Maria do Patrocínio Tenório Nunes

**Affiliations:** 1 Hospital das Clínicas da Faculdade de Medicina, Universidade de São Paulo, São Paulo, Brasil.; 2 Faculdade de Medicina, Universidade de São Paulo, São Paulo, Brasil.; 3 Faculdade de Medicina, Universidade do Porto, Porto, Portugal.

**Keywords:** Physician-Patient Relations, Patient Satisfaction, Medical Ethics, Health Communication, Medical Informatics, Relações Médico-Paciente, Satisfação do Paciente, Ética Médica, Comunicação em Saúde, Informática Médica, Relaciones Médico-Paciente, Satisfacción del Paciente, Ética Médica, Comunicación en Salud, Informática Médica

## Abstract

This study aims to evaluate the impact of the search for internet health information by patients on the doctor-patient relationship and satisfaction of both physicians and patients. In total, 200 patients of a tertiary hospital in Brazil and a convenience sample of 92 physicians were included. The questionnaires applied consisted of statements concerning the study’s objective; answers were given on a 5-point Likert scale. We conducted a descriptive analysis and used nonparametric tests to verify statistical differences in perception between subgroups of interest. In this study, 85.6% of internet users searched for internet health information but were skeptical about the information they found. They were mostly positive about the doctor-patient relationship; however, they desired greater engagement in health decisions. Overall, physicians tended to have a slightly positive view about internet health information impact on patients’ health despite some possible harmful effects. However, they believe that search for internet health information causes unnecessary fear and concern in patients, reduces doctor’s work efficiency, and that internet health information is not accurate or reliable. The massive search for internet health information has led to significant changes in the doctor-patient communication model. Both parties have demands to be addressed: patients need more reliable information; and physicians must adapt to these changes in a way that neither diminishes their autonomy as healthcare providers nor worsens the doctor-patient relationship. Hopefully, physicians could play a central role in educating patients and indicating adequate sources of information.

## Abstract (pt)

Este estudo tem como objetivo avaliar o impacto da busca por informações de saúde na internet por pacientes na relação médico-paciente e na satisfação de médicos e pacientes. Ao todo, 200 pacientes de um hospital terciário no Brasil e uma amostra de conveniência de 92 médicos fizeram parte da amostra. Os questionários aplicados consistiram em afirmações sobre o objetivo do estudo; as respostas foram dadas em uma escala Likert de cinco pontos. Fizemos uma análise descritiva e usamos testes não paramétricos para verificar diferenças estatísticas de percepção entre os subgrupos de interesse. Neste estudo, 85,6% dos internautas pesquisaram por informações de saúde na internet, mas estavam céticos quanto às informações encontradas. Eles foram principalmente positivos sobre a relação médico-paciente; no entanto, eles desejavam mais engajamento nas decisões de saúde. No geral, os médicos tendem a ter uma visão ligeiramente positiva sobre o impacto das informações de saúde na internet na saúde dos pacientes, apesar de alguns possíveis efeitos prejudiciais. Por outro lado, eles julgam que a busca por informações de saúde na internet causa medo e preocupação desnecessários nos pacientes, piora a eficiência do trabalho do médico e que as informações de saúde na internet não são precisas ou confiáveis. A busca massiva por informações de saúde na internet resulta em mudanças profundas no modelo de comunicação entre médico e paciente. Há demandas de ambas as partes a serem atendidas: os pacientes deveriam receber informações mais confiáveis; os médicos precisam lidar com essa mudança de uma forma que não diminua sua autonomia como profissionais da saúde nem piore a relação médico-paciente. Esperamos que os médicos possam desempenhar um papel central na educação e indicação de fontes adequadas de informação.

## Abstract (es)

Este estudio tiene como objetivo evaluar el impacto de la búsqueda de información de salud en internet por parte de los pacientes en la relación médico-paciente y en la satisfacción de médicos y pacientes. En total, 200 pacientes de un hospital terciario en Brasil una muestra de conveniencia estuvo conformada de 92 médicos. Los cuestionarios aplicados consistieron en declaraciones sobre el propósito del estudio; las respuestas se dieron en una escala de tipo Likert de cinco puntos. Se realizó un análisis descriptivo, mediante la aplicación de pruebas no paramétricas para verificar las diferencias estadísticas en la percepción entre los subgrupos de interés. En este estudio, el 85,6% de los usuarios de internet buscaron información de salud en internet, pero se mostraron escépticos sobre la información encontrada. En su mayoría los participantes consideraron las informaciones positivas sobre la relación médico-paciente; sin embargo, manifestaron la intención de tener una mayor participación en las decisiones de salud. En general, los médicos suelen tener una visión ligeramente positiva del impacto de las información de salud en internet en la salud de los pacientes a pesar de algunos posibles efectos perjudiciales. Por otro lado, coinciden que la busca de información de salud en internet genera miedo y preocupación innecesarios en los pacientes, empeora la eficiencia del trabajo del médico y que las información de salud en internet no son precisas ni confiables. La búsqueda masiva de información de salud en internet provoca cambios profundos en el modelo de comunicación médico-paciente. Hay demandas de ambas partes que deben cumplirse: los pacientes deben recibir información más confiable; los médicos deben lidiar con este cambio de una manera que no disminuya su autonomía como profesionales de la salud ni empeore la relación médico-paciente. Se espera que los médicos puedan desempeñar un papel central en la educación y la indicación de las fuentes de información adecuadas.

## Introduction

The expansion of digital access along with the growing search for internet health information by patients is a reality in Brazil ^1^ ^,^ ^2^. The prevalence of this behavior is on the rise and has become significant among patients: approximately 87% of internet users seek health information on the web ^2^.

This prevalent and profound behavioral change has led to significant transformations in the doctor-patient relationship, as it establishes a new communication model between them ^3^ ^,^ ^4^ ^,^ ^5^. Until recently, medical paternalism prevailed in this connection: the physician was the sole holder of incontestable scientific knowledge, while patients had a passive and submissive stance. Nowadays, this relationship is more horizontal: patients search for internet health information since they desire a more active role in their health-related decisions ^6^.

In this paradigm shift, ethical questions should be considered to ensure harmony between the patient’s autonomy and freedom of choice concerning their health and the physician’s professional autonomy ^6^ ^,^ ^7^ ^,^ ^8^. 

Previous studies evaluating physicians’ perception on the impacts of internet health information have yielded contrasting results. Some studies have suggested that searching for internet health information may increase patients’ understanding of their medical conditions and treatments, potentially leading to improved health outcomes ^3^ ^,^ ^6^ ^,^ ^8^. Others have suggested an increase in healthcare costs, unnecessary patient fear and concern, and worsening of doctor-patient relationship ^6^ ^,^ ^8^. Patients may also feel that their inquiries about internet health information could cause conflict during medical visits; this may explain why few patients talk openly to their doctor about internet health information when compared to those who only search for internet health information but do not share it ^9^ ^,^ ^10^. 

Studies have also suggested that cultural, demographic, and socioeconomic factors are related to different perceptions of internet health information’s impact on the doctor-patient relationship ^8^ ^,^ ^9^. This study aims to evaluate patients’ and physicians’ perceptions of the search for internet health information. This analysis considers the Brazilian cultural and social context, identifying differences among subgroups categorized by patients’ health and socioeconomic characteristics and physicians’ professional and demographic characteristics. Nevertheless, discrepancies in internet access across population groups in Brazil could not be fully addressed in this study, as 87% of the interviewed patients reported having internet access; which is significantly higher than the 67% reported for the lowest social classes ^11^. 

In this context, health literacy is a key determinant of the effect that internet health information may cause over patients; whether it has beneficial or harmful impacts. 

Searching for internet health information is a reasonable parameter for measuring Health Literacy, which is defined by Nutbeam [1998, *apud* World Health Organization ^12^ (p. 10)] as “*cognitive and social skills which determine the motivation and ability of individuals to gain access to, understand and use information in ways which promote and maintain good health*”. More recently, the concept of Digital Health Literacy, or eHealth Literacy, was developed to address the complexities of the constantly evolving communication scenario in the digital era. World Health Organization (WHO) ^13^ defines Digital Health Literacy as the “*ability to seek, find, understand, and appraise health information from electronic sources and apply the knowledge gained to addressing or solving a health problem*”. This concept encompasses challenges such as evaluating the accuracy of online health information; patients’ difficulty in determining source reliability; the risks of online misinformation over health; and inequalities in access to the internet and telehealth services across different populations ^14^ ^,^ ^15^.

More specifically, Health Literacy can be categorized into three types: (i) basic literacy, which refers to skills necessary for everyday situations related to health issues such as reading prescriptions, appointments, and medication labels; (ii) interactive literacy, which includes more complex cognitive skills that enable patients to extract relevant information, derive meaning from it, apply it to changing health circumstances, and make decisions; and (iii) critical health literacy, which involves the most advanced literacy skills such as the ability to critically analyze information from various sources and use it to exert greater control over life events and situations that impact health ^16^ ^,^ ^17^. Both interactive and critical health literacy are important determinants of how internet health information may impact the patients that search for it. 

The quality of online information is also another reason for concern among practitioners, as patients often encounter alarmist or inaccurate content. This is a valid consideration, as the reliability of internet health information is one of the factors that determine whether the search for health information will benefit or harm patient health outcomes. A systematic review ^18^ on the quality of online health information found that 55.2% of reviewed articles reached an overall negative conclusion regarding information quality. Most sources of online health information (social media, websites, forums, or blogs) fail to meet basic premises regarding reliability, such as authorship, purpose, funding, conflicts of interest, sources of information, empirical evidence, and use of statistical data ^19^ ^,^ ^20^. The unregulated nature of online information and its viral dissemination may produce many anecdotal absurdities, such as the case of an Australian wellness blogger who falsely claimed to have a terminal cancer that was refractory to radiation and chemotherapy but she successfully managed it with diet, exercises, and alternative therapies ^21^. She launched a mobile app with her miraculous treatment that was downloaded 200,000 times within the first month and earned her about USD 330,000. However, two years later, she admitted the cancer story was a complete fabrication ^21^. 

On the other hand, doctors are increasingly participating in this communicational dispute: more than 65% of physicians use social media for professional purposes ^22^. Social media offers a greater reach than traditional publication methods ^23^; thus, these professionals can play an important role in patient education and engagement when they are committed to sharing scientific relevant information, promoting health, and demystifying misinformation on a larger scale than was possible decades ago ^24^. However, there is also reason to be cautious about physicians spreading information on social media due to many reasons: professionals may have hidden economic goals; having a medical degree does mean that someone possess up to date, accurate, and reliable information, as many physicians recklessly spread misinformation on social media during COVID-19 pandemic ^25^. This phenomena may be explained by some principles of social media logic such as popularity - in which being “likeable” derives from a person’s ability to attract crowds rather than the veracity of the content - and connectivity or socio-technical affordance of networked platforms to connect users and advertisers via digital content, which may also influence the kind of content that gets more attention and views from users according to the platform algorithm. Again, it may have nothing to do with the quality of the information but rather from the capacity of engaging users by the content creator. In this new communicational model, the idea of sender, message, receiver, and channel, as it used to exist between physicians and patients, is clearly outdated. Now, between content creator and receiver, there are other actors involved such as developers, advertisers, and researchers - all influenced by algorithms that prioritize content that generates more engagement, likes, views, and revenue ^26^ ^,^ ^27^.

A solution for that should rely on evidence-based medicine, which can be defined as the conscientious, explicit, judicious, and reasonable use of current best evidence in making decisions about the care of individual patients ^28^. Evidence-based medicine has gained ground among practitioners in the last decades, as it has been shown to deliver better health patient outcomes. Nevertheless, we should not consider evidence-based medicine as an abstract and simplistic solution to a complex informational and communicational issue. Instead, it should be seen as a way of practicing Medicine that requires new skills from healthcare providers, such as the ability to evaluate clinical literature, apply it appropriately, and engage patients in the importance of prudent and reasonable use of information. 

Considering the panorama depicted above, this study aims to evaluate the impact of patients’ search for internet health information on the doctor-patient relationship: how this new informational scenario forces transformations on the dynamics between physicians and patients; its impact on quality of care; the conflicts that may emerge in this more horizontal interaction; the impact over healthcare costs and time efficiency during medical appointments; the perceived reliability of internet health information (in doctors’ and patients’ view) and its influence over patients’ health; and the satisfaction of physicians and patients in this scenario.

## Materials and methods

### Location of the study

The study was conducted at the Central Institute of the Clinics Hospital of São Paulo University (HC-FMUSP, acronym in Portuguese), a tertiary university hospital integrated into the Brazilian Unified National Health System (SUS, acronym in Portuguese). It is one of the largest hospitals in Latin America, in which almost 2 million outpatient appointments are conducted every year with a heterogeneous mass of people treated by multiple medical specialties. The hospital has about 21,600 employees of various professions and 31 medical specialties. 

### Patient sample size

Using a technique that estimates sample size based on prevalence studies ^29^, the minimum number of interviews was estimated considering the hypothesis that 87% of internet users search internet health information ^2^, as this data is a key aspect of the relevance of this study, using the following formula:

$$\text{n=}\frac{\left(z^{2}\right)P\left(1-P\right)}{d^{2}}$$

In which: *n* means sample size; *z* = z statistic for 95% confidence level (*z* = 1.96 for 95% confidence level); *P* = expected prevalence; and *d* = allowable error (5%). Consequently, a sample size of 174 patients was estimated.

### Eligibility and consent

This study included adult patients, aged 18 years or older, literate (able to read and write regardless of schooling degree), and without cognitive impairments that could interfere with questionnaire comprehension. To assure heterogeneity in the sample, no other eligibility criteria were required. Signed informed consent was obtained from all respondents before the interview. 

No eligibility criteria were required of doctors.

This study was approved by the Ethics Committee for Research Projects Analysis (CAPPesq HC-USP, CAAE: 14382819.5.0000.0068). All participants, physicians, and patients were duly informed of the research objectives and agreed to participate by signing a term approved by the Ethics Committee. The confidentiality and anonymity were secured for the participants.

### Study design

For patients, a convenience sample was adopted, including patients of diverse medical specialties. They were approached as they were waiting at the hospital for their regular appointment with doctors. They were asked to voluntarily answer a 10-minute paper questionnaire. Then, they were instructed about the Likert scale and left to answer the questionnaires by themselves. After the questionnaire was filled out, the data collector returned to pick it up. Interviews were conducted from August to October 2019 and took about 60 hours of work by the authors. An additional focus group of 44 random patients was interviewed about the source of internet health information they used to search.

For the physicians, convenience sample was also adopted. The data collection method was Computerized Self-Administered Data Collection using an online questionnaire that was sent to the doctors responsible for the clinics at HC-FMUSP, who were asked to forward it to assistant and resident doctors of their specialties. A total of 92 doctors answered the questionnaire.

### Research instruments

Patients answered a 26-item questionnaire, which consisted of 12 questions related to personal data and internet usage and 14 statements concerning internet health information and its impact on doctor-patient relationship according to a 5-point Likert scale (1 meaning “totally disagree” and 5 meaning “totally agree”). This Brazilian Portuguese questionnaire was adapted from a previous study ^9^ and additional questions and personal data were added with the goal of identifying other potential factors that may influence patients’ opinions and their source of information. The validation of this questionnaire, originally created in Portuguese, was performed in the reference study with 20 patients and its comprehensibility and intelligibility was validated. 

Physicians received a 37-item questionnaire, translated from English to Brazilian Portuguese, and adapted from a previous study that investigated doctors’ view about search for internet health information and its consequences ^8^. The translation and adaption were validated with a distinct group of 15 doctors that claimed total comprehensibility and intelligibility regarding the translated questionnaire. Also, personal and professional information were collected.

### Statistical analysis

Patients’ responses to questionnaires were transcribed into a spreadsheet of Microsoft Excel 2010 (https://products.office.com/); doctors’ responses were collected via Google Forms (https://docs.google.com/forms/), so they were imported and formatted in Excel. Data anonymization was performed to both groups. 

Subsequently, data was exported to a spreadsheet where statistics and tables were developed. Statistical tests were performed using the free software R, version 3.6.1 (http://www.r-project.org), under GNU General Public License v2. Moreover, R package *Rcmdr* and plugin *Rcmdr.EZR* were utilized.

#### Chi-squared independence test

Using both patients’ and doctors’ responses, we constructed cross tables with variables of personal data for the purpose of identifying possible confounding factors. Chi-squared independence test was applied and dependent variables were identified in each of these tables.

#### Kruskal-Wallis test

This nonparametric distribution free test was employed to perform comparisons between distributions of semiquantitative variables, that is, responses to statements on a Likert scale ^29^ ^,^ ^30^. It enabled the identification of statistically significant (p < 0.05) differences among subgroups of patients, divided by health condition, demographic characteristics, and internet behavior, and subgroups of doctors, divided by professional and personal data.

## Results

### Evaluation of patients

We interviewed a total of 200 patients. The sample was very heterogeneous regarding most personal data items, but some aspects should be highlighted: 72.5% of interviewed patients took continuous-use medication; 23.5% declared to be undergoing treatment for anxiety or depression; 61.5% were female; 79% showed a higher schooling level (high school or college); 81% exclusively used SUS as their health service; and 87% were internet users, of which 85.6% search for internet health information. Of those who search for internet health information, 47% have asked their doctors about internet health information. 

**Figure 1:**
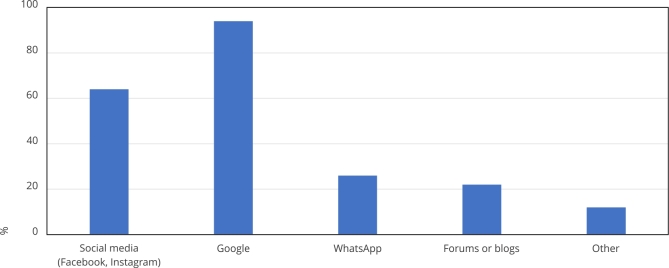
Patients’ digital information sources. Main sources of patient research on health in Brazil, in a random sample of 44 patients.

Their main source of internet health information was Google (93.18%), in which we found that one out of five Google users resort solely to the first link that appears in their research. Social media, such as Facebook and Instagram, are used by 63.64% of patients to search for internet health information. Messaging apps, including WhatsApp and Telegram, are used by 27.27% of patients; meanwhile, 22.73% reported using forums and blogs, and 11.36% reported using other resources (Figure 1).

Female patients search for internet health information more than male patients (p = 0.022). Higher schooling level, even when accounting for equal digital access, is associated with more search for internet health information (p = 0.029). Patients that have chronic diseases talk to their doctor about internet health information more often than non-chronic patients (p = 0.006). Patients treating anxiety or depression might be more likely to search for internet health information (p = 0.154) and may approach their doctors more often with information they obtained on the internet (p = 0.078). 

According to Table 1, in general, patients evaluated doctor-patient relationship positively. However, those who search for internet health information judge it more negatively and do not feel as if they have enough time to make questions during their appointments (p = 0.016). The respondents expressed a significant desire for more engagement in decisions about their health, female patients more often than male patients (p = 0.0001). Patients also demonstrate skepticism about internet health information, with only 24.5% judging it trustworthy; patients with higher schooling level and women trust internet health information even less (p < 0.05). This fact may be associated with patients’ desire for their doctors to indicate reliable sources of internet health information; those who search for internet health information (p = 0.018) and those who present internet health information to their doctors (p = 0.072) express this desire more clearly than the others. Even though more than one third of patients declare that their doctors tend to get dissatisfied after they present internet health information, that conflict tends occur, and that they do not feel comfortable approaching their doctors with internet health information, many of them still do it because they feel like they receive more attention and a better explanation from their doctors. Those with higher schooling levels feel their doctor gets dissatisfied when they show internet health information (p = 0.003), while those with lower schooling level perceive they receive more attention and a better explanation (p = 0.001).

**Table 1:** Patients’ responses about doctor-patient relationship and internet health information.

Questions	n	Totally disagree	Partially disagree	Neutral	Partially agree	Totally agree
%	%	%	%	%
I feel that my doctor listens to me	13	2.0	4.0	16.0	21.5	56.5
I feel that my doctor gives me enough time for my questions	14	3.0	8.5	12.0	22.5	54.0
By the end of the medical visit, I feel that I received satisfactory information about my health	15	2.0	4.0	13.5	21.5	59.0
I wish to be more engaged in decisions that concern my health	16	9.0	3.0	21.5	22.0	44.5
I wish my doctor would indicate to me reliable sources of Internet Health Information	17	13.5	2.5	18.5	16.5	49.0
I trust health information available on the internet	18	12.0	27.0	36.5	14.5	10.0
I am satisfied with health information I find on the internet	19	8.5	17.5	27.0	20.5	11.5
When I present internet health information to my doctor, I feel that he reacts satisfied	20	15.0	16.5	33.0	11.0	5.0
When I present internet health information to my doctor, a conflict does not occur	21	13.0	13.5	30.5	10.0	12.0
I feel comfortable presenting internet health information to my doctor	22	19.0	13.5	18.0	9.5	19.5
I feel that internet health information enables me to view my doctor as an equal	23	20.5	13.0	23.0	13.0	10.0
When I present internet health information to my doctor, he pays more attention on me	24	15.0	10.5	30.5	11.5	11.5
When I present internet health information to my doctor, our relationship is benefitted	25	14.0	11.5	30.5	11.5	11.0
When I present internet health information to my doctor, I feel that I receive a better explanation about my illness	26	7.0	9.0	24.5	14.0	24.5

### Evaluation of physicians

We received responses from 92 physicians. While heterogeneous in many aspects such as age, gender, and work shift, the sample concentrates responses from doctors from the Southeast Region of Brazil (79.3%) and who see less than 100 patients per week (78.3%). Subspecialist doctors comprise most of the sample (71.7%), while the rest are resident or general physicians; 72.8% work on clinical subspecialties. Physicians’ views about the impact of internet health information on patient’s health and doctor-patient relationship are both negative and positive, as shown in Table 2.

**Table 2:** Physicians’ responses about doctor-patient relationship and internet health information.

Questions	n	Totally disagree	Partially disagree	Neutral	Partially agree	Totally agree
%	%	%	%	%
Internet health information benefits people in the general sense	12	4.3	28.2	18.4	42.3	6.5
The exposure to personally relevant internet health information increases the patient’s sense of confidence and control during interactions with their physician	13	5.4	25.0	14.1	40.2	15.2
Internet health information encourages patients to follow treatment instructions or advice from their physicians	14	13.0	32.6	22.8	20.6	10.8
Internet health information contributes to rising the healthcare cost	15	4.3	15.2	18.4	35.8	26.0
Internet health information promotes unnecessary fear or concern about their health	16	3.2	7.6	4.3	47.8	36.9
Internet health information encourages patients to have more treatments of currently under-treated conditions	17	5.4	10.8	19.5	50.0	14.1
Internet health information improves people’s understanding of medical conditions and treatment	18	3.2	25.0	15.2	43.4	13.0
Internet health information causes patients to take up more of their physician’s time	19	5.4	17.3	14.1	40.2	22.8
Internet health information promotes unnecessary visits to physicians	20	7.6	19.5	13.0	36.9	22.8
Internet health information hinders doctor-patient relationship	21	17.3	30.4	16.3	26	9.7
Internet health information tends to be accurate	22	35.8	47.8	7.6	7.6	1.0
Most patients are able to judge the relevance of internet health information for their conditions	23	43.4	36.9	7.6	9.7	2.1
How relevant is internet health information to the patient?	24	8.6	13.0	38.0	34.7	5.4
How accurate is internet health information?	25	8.6	39.1	47.8	4.3	0.0
Do you have enough time to discuss internet health information?	26	16.3 No	83.6 Yes			
Do you feel that patients obtaining internet health information will be able to effectively manage their medical condition?	27	48.9 No	51.0 Yes			
Have you ever felt that a patient was challenging your authority?	28	41.3	15.2	29.3	5.4	8.6
Have you ever considered a patient’s request appropriate for their health?	29	55.4 No	44.5 Yes			
Did you attend the patient’s request?	30	34.8 *	57.6 **	7.6 ***		
Have you ever felt you know the patient well enough to have good communication?	31	20.6 No	79.3 Yes			
General perception						
Doctor-patient relationship	32	7.6	23.9	40.2	23.9	4.3
Time efficiency	33	7.6	32.6	46.7	13.0	0.0
Quality of care	34	3.2	16.3	38.0	36.9	5.4
Health outcomes	35	6.5	21.7	38.0	28.2	5.4

* No;

** Partially;

*** Completely.

As a positive point, they see the search for internet health information beneficial to patients’ health, as it is a way for them to prevent undertreatment and underdiagnosing of their diseases and it may be a tool for increasing their own comprehension about their health condition. As a negative point, 84.8% of physicians deemed that internet health information may cause unnecessary fear and concern, 80.4% claim that their patients are not able to judge internet health information’s relevance to their conditions, and 83.7% consider that online information is not accurate and reliable. Additionally, 45.7% of doctors consider that internet health information can worsen adherence to treatments proposed by them and 63% affirm that when patients search for internet health information and present it during the medical visit, it decreases the time efficiency of that appointment and increases healthcare costs. On average, physicians perceive that, on 36.5% of visits, patients ask them about internet health information and the main reasons for that are: to ask for their opinion about the information (62%), to request a diagnostic test (51.1%) or treatment (33.7%), and to demand changing their medication (13%). While 55.4% of doctors consider patient’s requests inappropriate in regard to their health condition, only 34.8% reject these requests, with the majority accepting it partially (57.6%) or completely (7.6%).

Particularly, professionals that see more than 100 patients per week (p = 0.009) and women (p = 0.031) have a more negative view about internet health information impact over patient’s health and adherence to treatment. Moreover, unlike from the rest of the sample, doctors aged 30 or less reported feeling that internet health information makes patients demand more time of them (p = 0.058); they reported feeling that consultations lack time to discuss this information (p =0.0014) and that their medical authority is challenged (p = 0.021). 

Surgeons believe their patients are less apt to judge internet health information’s relevance to their conditions than clinicians (p = 0.038); clinicians felt they knew the patient well enough to have good communication in case they bring up internet health information during the medical visit (p = 0.026). 

## Discussion

Patients’ results in our study are consistent with the 2014 publication by McDaid & Park ^2^, which has indicated a very high prevalence of search for internet health information that has increased in comparison to older studies ^3^ ^,^ ^6^ ^,^ ^10^. Despite their search for internet health information, most of our interviewed patients reported a good doctor-patient relationship, revealing the same pattern present in a previous study ^9^. However, this positive perception over the relationship should not be generalized to every social and cultural panorama without further investigation. Even in this study, some subgroups (patients with lower schooling levels and those with chronic diseases) tended to have a more positive opinion compared to others (patients with higher schooling levels). Furthermore, on the contrary to the generally positive relationship described in this study, medical litigation is has been on the rise, increasing 176% in median damage award from 1994 to 2001 ^31^. Indirectly, we assessed this concern between our physicians verifying the practice of defensive medicine, which includes ordering unnecessary exams tests, to attend patients’ desire and avoid conflicts. According to this definition, defensive medicine was practiced by 65.2% of our interviewed doctors. 

We observed that internet health information has some positive impact over healthcare and doctor-patient relationships and negative influence over other aspects such as cost, time efficiency, and adherence to treatment. Overall, according to Freckelton ^32^ (p. 502), “*the ubiquitous access by patients to online information about health issues is disrupting the traditional doctor-patient relationship in fundamental ways*”.

We observed that a significant proportion of patients who present internet health information to their doctors perceive a conflict emerging and even a tense environment during their medical visits. Our professionals who attend more than 100 patients/week, surgeons, women, and doctors under 30 have negative opinions about internet health information. On the other hand, this study gathered new insights about what patients’ subgroups are more likely to search for internet health information (women, patients with higher schooling levels, and those with chronic diseases). 

Those previous observations bring a paradox to be addressed: although searching and discussing internet health information may be an important tool for empowering the patient, making them health literate and promoting better health outcomes; this attitude comes with the burden of a conflict with the doctor. 

According to Newnham et al. ^33^, 40% of patients felt the doctor-patient relationship was unaffected by information searching, 24% felt it improved the relationship, and only 8% felt it adversely affected the relationship. 

Tan & Goonawardene ^34^, in a systematic review, found eight studies that examined factors that directly affected doctor-patient relationship. Most patients believed that internet health information seeking did not adversely impact the relationship with their physicians. The positive effect of online information was stronger when patients had an opportunity to discuss the information they found online; and when physicians did not look challenged by information brought by patients. Moreover, bringing internet health information was found to have a more positive influence when physicians displayed adequate communication skills in discussing patients’ queries and when both parties had a good prior relationship. 

Despite our study showing a limited prospective approach, we investigated the expected role of the physician in this new communication scenario, that is, the role of indicating reliable sources of internet health information and educating their patients about how to deal with and filter these information. A solution to this issue is to ally health literacy, and consequently healthier patients, with a more solid doctor-patient relationship. Internet health information search behavior empowers patients to play a more active role in their disease management, enabling them “to do something” rather than “just being told what to do” by their specialist. Internet search provides clarity in terms of treatment options, but on the other hand, may diminish patients’ reliance on their specialists ^34^.

Our physicians were more optimistic about the effect of internet health information than the respondents of the 2009 reference study conducted by Kim & Kim ^8^. Nevertheless, the same concerns were found among our respondents: low reliability of internet health information, patient’s inability to judge its relevance, damage on time efficiency, increase in healthcare costs, and decrease in treatment adherence. On the other side, our respondents mentioned a higher prevalence of patients desiring to interfere with the conduct adopted by the health professional, such as demanding a diagnostic test, a different treatment, or requiring change of medication. 

Patients’ sense of empowerment is dependent on how receptive providers and specialists are to patients’ desire to take part in the decision-making process ^35^. Regarding patients’ compliance with their treatment, both physician quality (competence, empathy, and communication) and internet health information quality have significant impacts on patient-physician concordance, with physician quality exhibiting a much stronger relationship ^36^.

Our doctors also claimed, in a significantly greater proportion, to have had enough time to discuss internet health information and to know the patient well enough to establish good communication when compared with the cited study ^8^. The observed negative view about internet health information impact over doctor-patient relationship by those who see more than 100 patients per week enlightens the deleterious consequences of a heavy workload over the medical practice and the health outcomes to the patient.

### Limitations

Interviews with patients were conducted by medical students in the hospital while patients were waiting for their medical appointments, which may have inhibited more critical responses about doctor-patient relationship. On the other hand, patients filled the questionnaires by themselves without the presence of any observer. 

As this study was conducted in a university tertiary hospital, using a convenience sample, it may show limitations for generalizing results. However, the heterogeneity of the sample regarding personal data mitigates this possible bias. 

When a patient uses a search engine (i.e., Google), there is a limitation to evaluate the kind of content that is really accessed; it may be a layman website, a website with commercial purpose, a doctor’s website filled with scientific information, etc. Thus, by now, it is difficult to measure the quality of the information that a patient obtain via search engines. This specific point was not approached in the present study.

## Conclusions

In summary, this study shows patients’ and physicians’ perception of internet health information and its impact over doctor-patient relationship. It confirms some key findings from previous studies: patients’ search for internet health information is a very prevalent behavior; physicians judge internet health information unreliable; and internet health information impacts doctor-patient relationship. This study also adds new insights about the investigated matters: patients demonstrate skepticism about internet health information and want their doctors to indicate reliable sources of information; young physicians have more difficulty to deal with internet health information because they feel it is harder to establish good communication and it worsens time efficiency of the medical visit; female physicians and those who see more than 100 patients per week have a more negative view about internet health information impact on patients’ health and doctor-patient relationship.

It is undeniable that searching for internet health information is a tool for empowering patients and promoting their autonomy and what is coherent to their own desires. On the other hand, potentially harmful consequences and challenges of that behavior may arise if this new communication model is not adequately managed and if internet health information is inaccurate. In this sense, doctors should try to bring evidence-based medicine to their daily practice and also to transmit to patients the attributes of accurate health information. Doctors can advise their patients that their main sources of information (Google, social media, and messaging apps) have weak or no policies of verifying the reliability and quality of this information. It is important to consider that this kind of conversation can only be imagined in a frank, open, trustful relationship between doctors and patients. It would be naive to expect that patients would passively accept their doctors advice regarding sources of information and also that doctors would not feel minimally challenged or dissatisfied when a patient bring an information acquired via social media, for example. Nowadays, internet and social media play a central role in people’s everyday life, in their feelings, their affections, and their values, also influencing their interpersonal interactions, including in their medical appointments. If doctors are capable of treating it as a matter of fact, and not only as a motive for dissatisfaction or conflict, a different communicational and relational bond can be established based on the same millenary ethical principles that guide physicians toward their ultimate goal of providing the best healthcare for their patients: autonomy, beneficence, non-maleficence, and justice. 

Clearly, this much more complex atmosphere permeated by massive quantities of information, influenced by mass media personas who lead crowds of people and contaminated by any kind of misinformation, demand a deep reflection from physicians. Rethinking the old information asymmetry between them and their patients and developing communicational skills that enable them to dialogue in a frank and constructive manner can be a path to promote a better health care in the digital era.

We should remember that, in many cases, as expected, patients will not have total capability of judging the accuracy of what they find on the internet. In this case, practitioners may try to provide them enough information during the medical appointment and also, when needed, to present them good sources of information with evidence-based information such as UpToDate. Unfortunately, most of the reliable evidence-based information apps and sites are designed and available only to health practitioners and are not adapted to patients such as MedScape, Essential Evidence Plus, and DynaMed. 

The COVID-19 pandemic showed a fast spread of questionable information, disseminated even by doctors via online platforms. Such information negatively impacted health outcomes, costed human lives, and also caused a significant resource waste due to unproven therapies ^25^. Thus, it is clear that we are living a perfect moment for health providers, companies, and governments to expand the access to evidence-based information to patients as a cost-effective way of promoting health. In this process, we should consider well-established guidelines and validated sets of criteria for judging the quality of health information written for the public such as DISCERN ^37^ or JAMA ^38^ in order to assure the quality of the information.

As this study reveals, searching for internet health information is not caused by a deteriorated doctor-patient relationship, conversely, those who adopt this attitude are the most eager to hear from their doctors’ trustworthy sources of health information. Patients’ skepticism about internet health information reliability is a topic that doctors may play a central role, aiming towards health education. This process should not be considered by physicians as mere information transfer; it also includes having a positive attitude toward counseling, empowering the patient to make decisions based on scientific evidences, and establishing a positive bond that promotes healthy behavior changes.

Although this is a cross-sectional study that depicts a broader panorama over patients’ behavior towards health information and its impact over the doctor-patient relationship, some insights may orient necessary reflections over medical practice. Healthcare providers must understand that the search for internet health information is a very prevalent posture, and they should try to comprehend patients’ motives in order to establish a better relationship with them. According to our findings, inquiring doctors about internet health information can be a way of the patient getting more attention and better explanation over their health condition. 

Lastly, concerns about healthcare costs and time efficiency are reasonable and real. Nevertheless, interventions on health education in this new communication model pervaded by internet health information should be analyzed considering its time and cost-effectiveness as it is clear that a better informed patient tends to have improved health behaviors and health status.
